# When a Placebo Is Not a Placebo: Problems and Solutions to the Gold Standard in Psychotherapy Research

**DOI:** 10.3389/fpsyg.2018.02317

**Published:** 2018-11-26

**Authors:** Cosima Locher, Jens Gaab, Charlotte Blease

**Affiliations:** ^1^Division of Clinical Psychology and Psychotherapy, Department of Psychology, University of Basel, Basel, Switzerland; ^2^Program in Placebo Studies, General Medicine and Primary Care, Beth Israel Deaconess Medical Center, Harvard Medical School, Harvard, MA, United States; ^3^School of Psychology, University College Dublin, Dublin, Ireland

**Keywords:** Psychotherapy, placebo, clinical trials, common factors, open-label placebos, ehealth, control conditions

Randomized placebo-controlled trials are recognized as the gold-standard of evidence-based medicine but when it comes to psychotherapy research all that glitters is not gold. Translation of this standard from medicine to clinical psychology is fraught with difficulties. While a wealth of robust evidence shows that psychotherapy is effective for a range of mental health conditions the use of placebo controls to assess the effectiveness of specific psychological interventions faces serious conceptual and methodological challenges (Gaab et al., 2018).

In this Opinion article we identify two under-appreciated placebo-related problems which substantially risk the validity of clinical trials in psychotherapy. The first is a common misconception about the nature of placebos; the second is the problem of double-blinding. We review current solutions and future prospects for the gold-standard in psychotherapy research.

## What are placebos?

In clinical research placebos usually refer to methodological devices employed to investigate the specific effectiveness of a treatment. While it is seductive to define placebos as “things” e.g., “sugar pills” in pharmacological trials and “attention controls,” “active controls” or “non-directive controls” in psychotherapy trials, placebos are more correctly conceived as instruments used for measuring the efficacy of a treatment. As such they should be understood as a moving category (Blease, 2018b). In every randomized controlled trial (RCT) the placebo should ideally be “bespoke” – tailored to mimic an intervention under investigation without consisting of any it's hypothesized characteristic constituents. While fulfilling this goal is enormously challenging, in drug trials a placebo should preferably mimic the particular taste, appearance, and method of administration of the specific intervention without comprising the treatment's characteristic pharmacological ingredient(s). Placebos should also ideally be administered double-blind: practitioners/researchers and patients should not be able to distinguish the placebo from the treatment.

We also point out that double-blinding is not inherently associated with the term placebos in clinical research. A second, nuanced and different use of the term comes under the label “open-label placebos” (OLPs) (Blease, 2018b); here “placebo” refers to specific interventions (usually sugar pills) which are administered alongside other socio-emotional cues in the patient-practitioner encounter usually with the aim of eliciting salubrious placebo effects (Sandler and Bodfish, 2008; Kaptchuk et al., 2010; Carvalho et al., 2016; Schaefer et al., 2016; Locher et al., 2017). This distinctive interpretation of “placebos” is not our focus.

Here we are interested in placebos as controls in RCTs. We argue that while formulating suitable placebos and double-blinding conditions is a challenge in pharmacological trials the task is even more formidable when it comes to testing psychological treatments.

## What is the problem with placebos in psychotherapy trials?

Different psychotherapy interventions have their own highly characteristic treatment constituents derived from divergent psychological theories. Beyond their characteristic constituents, however, different approaches typically share a variety of so-called “common factors” including an empathetic practitioner; a rationale behind the version of therapy; provision of positive cues by the practitioner; and expressions of hope about the patient's outcome, all of which are considered necessary if not sufficient for the effectiveness of talking therapies (Cuijpers et al., 2012). Regardless of their disputed potency, “common factors” present the first major challenge with placebo-controlled trials in psychotherapy since adequate placebos need to control for (that is to say, mimic) these variables without also applying the characteristic techniques of the intervention under evaluation. Manifestly, this measure is not typically applied in psychotherapy research where the quality of waitlists or controls is rarely interrogated or systematically reported by researchers.

The second, related problem is double-blinding which requires controlling for both patient and researcher/practitioner expectations. Given that psychotherapy relies on verbal, interpersonal interaction it seems impossible to implement placebos in clinical research without patients and clinicians being fully aware of the true nature of their treatment allocation. It is widely recognized that patients' perceptions about the credibility of therapists (and treatment methods) can mediate expectations about outcomes and increase the size of placebo effects. Failure to effectively control for patient expectations can thereby lead to exaggerated reports about characteristic treatment effects (Baskin et al., 2003).

Similarly, researcher allegiance in psychotherapy influences the outcome of treatments in profound and often subtle ways by shaping the operationalization of the treatment (e.g., better training of therapists in the preferred treatment) thereby affecting therapists' behavior and patients' experiences (Gerger and Gaab, 2016). Indeed, one recent, noteworthy meta-analysis reported that differences between psychotherapy treatments and “placebos” disappear after allegiance was controlled (Cuijpers et al., 2012).

In summary, inadequate conceptualization of placebos can undermine judgments about the relative effect size of the psychotherapy under investigation. This is also true where patients allocated to waitlist controls often experience worsening of symptoms (Furukawa et al., 2014) while those allocated to the treatment group experience benefits: too often this difference is hastily attributed to the treatment's specificity.

## What are the solutions?

Among those who have confronted psychotherapy's placebo-problem, a number of control group strategies have been proposed: First, waitlist controls, no-treatment or usual care should ideally control for threats to internal validity with respect to evaluating the intervention (Freedland et al., 2011), i.e., control for regression to the mean, spontaneous improvement of disorders and symptoms, as well as the Hawthorne effect (the effect of being observed and evaluated; Patterson et al., 2016). Recently, no-treatment and usual care have been proposed as the appropriate control for establishing the efficacy of a psychotherapy (Kirsch et al., 2016). However, it is well known that these passive control conditions lead to an overestimation of the efficacy of a psychotherapy (Mohr et al., 2014) and are inappropriate for the identification of specific treatment components (Gaab et al., 2016). Furthermore, having to wait for a treatment potentially hinders patients' self-healing behaviors and waiting list controls in RCTs may even contribute to nocebo effects (Furukawa et al., 2014; Cuijpers and Cristea, 2015, 2016).

In contrast, findings show that active control conditions such as “attention controls,” “active controls” or “non-directive controls” produce smaller *relative* effect sizes of interventions than compared to passive controls (Mohr et al., 2014). In active controls, the clinician-patient relationship (Kelley et al., 2014), trust in the clinician (Birkhäuer et al., 2017), as well as patients' expectations (Jepma and Wager, 2015) contribute to symptom relief (Lambert, 2013; Wampold and Imel, 2015). Similarly, it has been shown that the comparison between psychotherapy and structurally equivalent control conditions (i.e., control condition and active treatment are comparable on the following dimensions: number of sessions, length of sessions, format, training of the therapist, whether the intervention was individualized to the patients, and whether patients can discuss topics logical to the treatment) produce smaller effects than the comparison between psychotherapy and structurally unmatched control conditions (Baskin et al., 2003). In summary: the wide variation of control groups across studies seriously undermines the comparability of study results (Zhu et al., 2014).

Second, a way to address the question of *how* (and not *whether*) psychotherapy works is through trials that directly compare two or more treatments to each other (Marcus et al., 2014). Comparative trials–also labeled as “horse race” studies–assume that psychotherapies are generally effective, but aim to find out which type of therapy works best (Flückiger et al., 2018). However, it has been argued that especially in these trials, there is a large association of researcher allegiance to relative efficacy (Imel et al., 2008). Moreover, meta-analyses of comparative studies have not revealed substantial differences between psychotherapies (Spielmans et al., 2007; Benish et al., 2008; Imel et al., 2008; Miller et al., 2008) – a finding which is consistent with the Dodo Bird conjecture of treatment equivalence from 1936 (Rosenzweig, 1936).

A third approach is to conduct dismantling studies whereby the standard treatment is compared to another treatment without a characteristic constituent. On the premise that the treatment has been found to be effective, the logic of the design is to “dismantle” the treatment in order to identify its specific ingredients. Correlatively, “additive studies,” add a characteristic constituent to the standard treatment: on this perspective it is hypothesized that the added specific ingredient will augment the efficacy of the treatment (Ahn and Wampold, 2001). So far meta-analyses have found little evidence that full treatment packages are superior to partial treatment packages at the end of psychotherapy (Ahn and Wampold, 2001; Bell et al., 2013).

A fourth strategy aimed at tackling the problem of psychotherapy RCTs is illustrated by Kim et al.'s investigation (Kim et al., 2012) into breathing therapy–a widely used component of cognitive-behavioral therapy for patients with panic disorder–which directly compared two opposing psychotherapy treatments: breathing training to either increase or decrease end-tidal partial pressure of carbon dioxide among patients. Here, the usual RCT logic was inverted (i.e., instead of controlling the incidental constituents and manipulating the characteristic constituents, the characteristic constituents are held constant and the impact of the incidental constituents is examined). In this experiment, it was discovered that neither intervention was superior: the effectiveness of the interventions was interpreted by the authors to be attributable to the common factors in the delivery of the treatments. Although promising, there exists only one study so far (Kim et al., 2012).

Finally, a promising methodology is the so-called “open/hidden” administration of a given treatment. In drug trials this method entails giving a drug in full view of the participant (“open”) vs. without the participant's knowledge (“hidden”). Recently, an open/hidden design was incorporated into psychotherapy interventions. Two groups received the same online expressive writing intervention but different treatment rationales: participants in the open-arm were told that the intervention had beneficial effects on mood in the long-run whereas participants in the hidden-arm were informed that mood only influenced how they perform in the intervention. Significant differences were observed as shown by a long-term decrease of negative affect in the open-arm only (Tondorf et al., 2017). However, this study included healthy subjects and the design should still be tested in a clinical setting.

A significant limitation of many of these approaches is their unsuitability for investigating the myriad techniques that are dependent on therapist-patient interactions. Is there a way to overcome this hurdle? New technologies which combine affective computing with machine learning present unprecedented opportunities to “work around” the problems associated with providing “placebo” psychotherapy (Blease, 2018a). Innovations in eHealth and mHealth already challenge traditional assumptions about the delivery of psychotherapy and some commentators speculate that virtual humans and avatars may one day obviate the need for human therapists. While this research is still in gestation these technologies may better allow researchers to control for “common factors” in psychotherapy–for example, by standardizing levels of empathy and positive cues by avatar “therapists,” and eliminating researcher/therapist bias–thereby permitting appraisal of specific treatment techniques (Rollman et al., 2018). Although eHealth technologies have been discussed as an effective way to foster the active role of patients in their healthcare (Barello et al., 2016) as a preliminary step in testing the potential for e-therapy it would first be necessary to directly compare the effectiveness of standard psychotherapies (delivered by humans) with therapy delivered by virtual humans or avatars. Beyond these possibilities, many current internet interventions–including app-based interventions–make it easier to employ double-blind treatments since they avoid problems associated with inter-personal delivery of care.

## Conclusions and recommendations

We strongly advocate the use of innovative methodologies and eHealth technologies to overcome the conceptual and empirical hurdles of placebos in psychotherapy research–significant challenges that, we argue, too often go unrecognized. We conclude with three additional recommendations to help promote awareness of these issues with the goal of fostering scientific rigor in psychotherapy clinical trials. First, we advocate the need for “placebo-literacy” among investigators to encompass education about placebos in healthcare research (Blease et al., 2017). Second, we propose that psychotherapy trials be obliged to report comprehensive descriptions of all placebo control components including the rationale provided to patients, as well as structural criteria (i.e., number and length of sessions, format, and training of therapists; Baskin et al., 2003). Third, we argue that published articles should include a declaration of researchers' allegiance. Such steps are important to preserve the integrity of evidence-based practice (and avoid overestimation or underestimation of relative effect sizes); they also carry valuable ethical repercussions for patients: improved scientific understanding about the effectiveness of different psychotherapy interventions allows practitioners to provide patients with higher quality care, and more accurate disclosures about how treatments work.

## Author contributions

CL, JG, and CB conceived and designed the Opinion Paper. CL wrote the first draft of the manuscript. CL, JG, and CB wrote the final version.

### Conflict of interest statement

The authors declare that the research was conducted in the absence of any commercial or financial relationships that could be construed as a potential conflict of interest.
